# A Model of Generating Visual Place Cells Based on Environment Perception and Similar Measure

**DOI:** 10.1155/2016/3253678

**Published:** 2016-08-14

**Authors:** Yang Zhou, Dewei Wu

**Affiliations:** Information and Navigation College, Air Force Engineering University, Xi'an, Shaanxi 710077, China

## Abstract

It is an important content to generate visual place cells (VPCs) in the field of bioinspired navigation. By analyzing the firing characteristic of biological place cells and the existing methods for generating VPCs, a model of generating visual place cells based on environment perception and similar measure is abstracted in this paper. VPCs' generation process is divided into three phases, including environment perception, similar measure, and recruiting of a new place cell. According to this process, a specific method for generating VPCs is presented. External reference landmarks are obtained based on local invariant characteristics of image and a similar measure function is designed based on Euclidean distance and Gaussian function. Simulation validates the proposed method is available. The firing characteristic of the generated VPCs is similar to that of biological place cells, and VPCs' firing fields can be adjusted flexibly by changing the adjustment factor of firing field (AFFF) and firing rate's threshold (FRT).

## 1. Introduction

In 1971, O'Keefe and Dostrovsky [1] found that some neurons in rat's hippocampus exhibited location selectivity during rat free moving. They defined these cells as place cells. The activity of place cells is related to the spatial location. Once the animal is located at a relatively narrow region, the corresponding place cell can fire with a high rate. But in other regions, this place cell does not fire or fires with a low rate. This firing characteristic shows animal's spatial representation pattern and navigation mechanism to a certain extent, and it lays a biological foundation to the bioinspired navigation technology, for example, RatSLAM [2, 3], which is realized by simulating the navigation mechanism in hippocampus and it can provide the robot with a spatial awareness of entire environment.

How to generate place cells? Brain neuroscience points out that the firing of place cells can be activated by the idiothetic information (e.g., self-motion) and allothetic information (e.g., visual) [4–7]. For the idiothetic activity mode, it can be realized through the transformation from the grid cells to the place cells. The existing models include the following: transformation model based on Fourier analysis [8], transformation model based on competitive learning [9], transformation model based on independent component analysis (ICA) [10], and so on. For the allothetic activity mode, it is achieved mainly through the process of obtaining landmarks, place code, and the calculation of place cell's firing rate. This paper mainly focuses on the second-activity mode based on visual information, and the generated place cells are called visual place cells (VPCs) [11, 12]. Doboli et al. [13] proposed an attractor model of the hippocampus. In their model, the external sensory input encodes distances to perceived landmarks as well as allocentric bearings to them, and VPC's external receptiveness is formed by a product of Gaussian functions. Gaussier et al. [11, 12, 14, 15] had done a lot of work in the field of generating VPCs. In their researches, VPC is defined by a spatial constellation of online learned visual view, which is obtained by the process of gradient processing, convolved with a difference of Gaussian filter (DoG), selection of focus point, and log-polar transformation. During the learning of a location, each local view in log-polar coordinate is learned as a landmark for the system; then, the landmarks' recognition information and their spatial localization in the visual field are merged in a product space to define a place code, and the VPC is generated after the recognition and association of the place code. DalleMole and Araújo [16] proposed a topological map of place cells. To produce a cognitive topological map of the environment, they acquire and organize knowledge associating it with places and define an activation function to determine the similarity between the current perception, the stored memories, and so on.

Through the analysis of the current research status, we know that there is a great similarity in the existing VPCs' generation methods, and their differences mainly are reflected in the acquisition and representation of landmarks and the calculation of VPCs' firing rates. This paper abstracts a unified VPCs' generation model after fully considering their similarities and differences. The acquisition and representation of landmarks are included in the environment perception. The calculation of VPCs' firing rates is included in the similarity measure. As a result, the generation process of VPCs and the issues that need to be solved are stated more clearly, which can provide a complete research idea for the related researchers. Simultaneously, according to the abstracted model, a specific method for generating VPCs is presented, and it is validated and analyzed by the simulation.

## 2. VPCs' Generation Model

**Figure 1:**
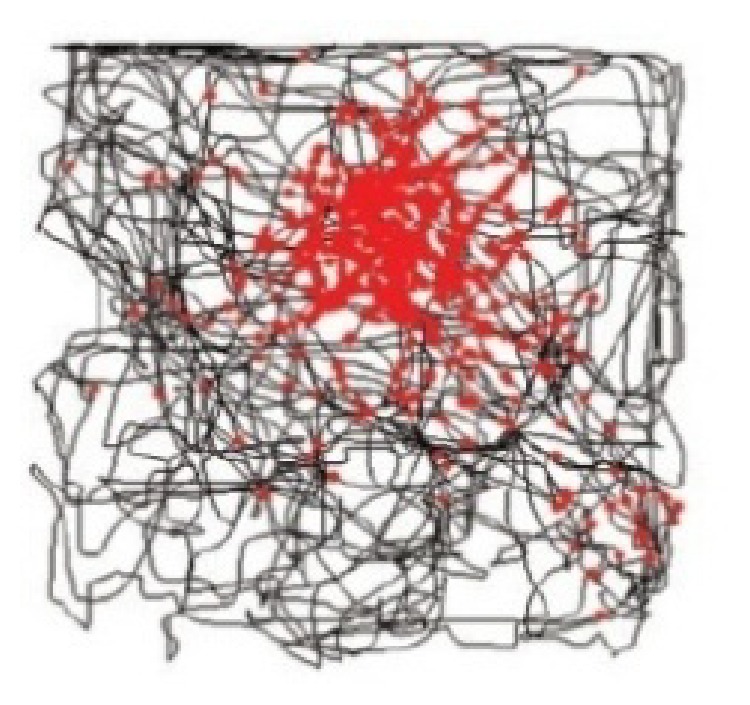
Firing pattern of place cells [17].

The firing activity of biological place cells exhibits strong location selectivity. When the animal is located in the region represented by place cells, the corresponding place cells will fire with high rate. In biology, the field with the firing activity is called “place field” or “firing field.” Figure 1 shows the firing activity of place cells recorded by biological experiment [17], where the trajectory of rat is indicated by gray line, and each spike is plotted in red. In the theory of hippocampal cognitive map, place cells are believed to constitute the basic unit of this cognitive map. A single place cell can represent a specific location in the environment, and the firing activity of the whole place cells can describe and represent the entire environment.

**Figure 2:**
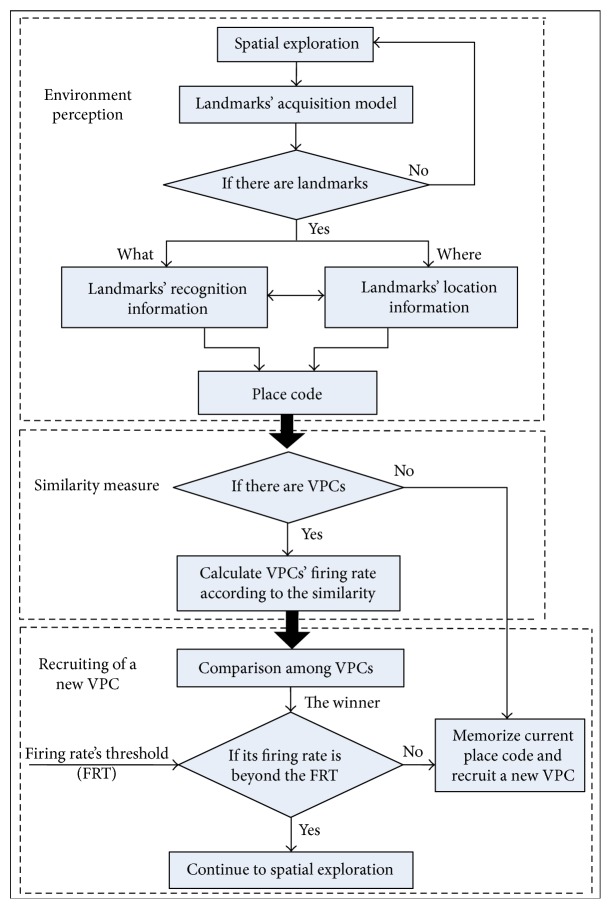
VPCs' generation model.

By analyzing and summarizing the existing VPCs' generation methods [10–16], VPCs' generation model is abstracted here based on environment perception and similarity measure, as shown in Figure 2. VPCs' generation process is divided into environment perception, similarity measure, and recruiting of a new VPC. The environment perception phase achieves the landmarks' acquisition and place code. Landmarks' acquisition resolves what information is used as reference and how to represent it. In this paper, a landmark is also described by two kinds of information as shown in [15]. One is the recognition information (What) and the other one is the location information (Where). What information and Where information are merged to get the representation information of a landmark. Place code is to define current location by the whole landmarks, and each generated VPC is associated with a place code. Similarity measure achieves the analysis of the similarity between current place code and the generated VPCs and then quantifies the VPCs' firing rates according to their similarity. The phase of recruiting a new VPC achieves the analysis of the firing status of the existing VPCs and flexibly recruits a new VPC.

VPCs' generation process can be summarized as follows: during spatial exploration, the vehicle acquires the recognition information and location information of landmarks by especial environment perception approach and gets the place code according the landmarks' representation information. Then, VPCs' firing rates are calculated according to the similarity between their associated place codes and current place code. Finally, the comparison among the generated VPCs is implemented and the winner is compared with the given firing rate's threshold (FRT) (FRT is set as the condition to recruit a new VPC; it indicates the minimum firing rate that the generated VPCs should satisfy). If it is below FRT, the current place code is memorized and a new VPC is recruited to be associated with this memory.

Next, a specific VPCs' generation method will be presented according to above-mentioned process; the details are as follows.

**Step 1 (acquire landmarks).**

Combining local invariant theory and our previous proposed model for landmarks' acquisition [18], the attention points which are obtained by the process of extraction of feature points, generation of saliency value, and selection of attention points in the visual image are used as landmarks. Considering we mainly focus on the generating VPCs, the landmarks' acquisition process is not discussed in detail here.

**Figure 3:**
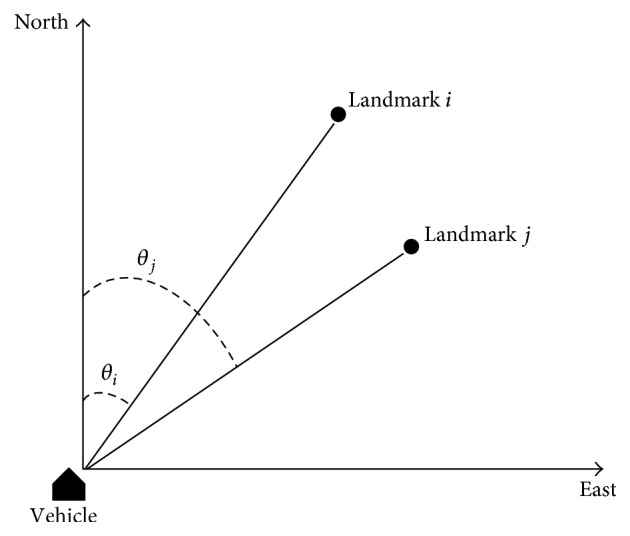
Schematic diagram of landmarks' orientation.

Let the number of acquired landmarks at current moment be *N*(*t*). The recognition information and the saliency value of landmark *i* are denoted by *r*_*i*_(*t*) and *s*_*i*_(*t*), respectively. The distance and the orientation of landmark *i* relative to the vehicle are denoted by *d*_*i*_(*t*) and *θ*_*i*_(*t*), respectively. Then, the landmark is represented as follows:

(1) $$\begin{array}{c} l_{i}\left(\;t\;\right)=\left(\;r_{i}\left(\;t\;\right),s_{i}\left(\;t\;\right),\theta_{i}\left(\;t\;\right),d_{i}\left(\;t\;\right)\;\right),\;i=1,2,\ldots ,N\left(t\right), \end{array}$$

where *l*_*i*_(*t*) denotes the representation information of landmark *i*. *θ*_*i*_(*t*) denotes the orientation between north direction and the vector from vehicle to landmark *i* in clockwise direction. The schematic diagram of landmarks' orientation is shown in Figure 3. In this paper, the absolute direction is used as the reference direction to calculate landmarks' orientation. The reason is that if the reference direction (such as vehicle's running direction) may change at different location or different time, then the calculated landmarks' orientation relative to this kind of reference direction may be affected by the change of reference direction, which finally makes it difficult to measure the accurate relation between landmarks and vehicle at the same location.

**Step 2 (construct place code).**

The whole representation information of landmarks at current moment is combined to get current place code, denoted by *C*(*t*); namely,

(2) $$\begin{array}{c} C\left(\;t\;\right)=\left\{\;l_{1}\left(\;t\;\right),l_{2}\left(\;t\;\right),\ldots ,l_{N\left(t\right)}\left(\;t\;\right)\;\right\}. \end{array}$$

**Step 3 (design similarity measure function to quantify VPCs' firing rates).**

In our paper, the similarity between VPCs' place codes and current place code is used to evaluate VPCs' firing rates. The similarity measure function is designed based on Euclidean distance and Gaussian function; the formula is as follows:

(3) $$\begin{array}{c} f_{k}\left(\;t\;\right)=\overset{N_{k}}{\underset{i=1}{\sum }}w_{i}e^{-\left[w_{d}\left({\left(d_{i}\left(t\right)-d_{i}^{k}\right)}^{2}/\sigma_{d}^{2}\right)+w_{\theta }\left({\left(\theta_{i}\left(t\right)-\theta_{i}^{k}\right)}^{2}/\sigma_{\theta }^{2}\right)\right]}, \end{array}$$

where *f*_*k*_ is the firing rate of VPC *k*. *N*_*k*_ is the number of matched landmarks between VPC's place code and current place code. *σ*_*d*_^2^ and *σ*_*θ*_^2^ are defined as the adjustment factor of firing field (AFFF). They denote the influence on VPCs' firing fields from the distance and orientation, respectively. The bigger the AFFF, the larger the firing fields with high firing rate. *w*_*d*_ = {0,1} and *w*_*θ*_ = {0,1} denote whether the distance and orientation are used as the condition of similarity measure, respectively. If the answer is yes, its value is set to one, otherwise it is set to zero. *w*_*i*_ denotes the contribution to calculating VPCs' firing rates. *w*_*i*_ can be calculated by the saliency value of landmarks. The formula is as follows:

(4) $$\begin{array}{c} w_{i}=\frac{s_{i}}{\sum_{i=1}^{N\left(t\right)}s_{i}}. \end{array}$$

From (4), we know that the bigger the landmark's saliency value (that is to say, the better the landmark's robustness), the bigger its contribution to calculating VPC's firing rate.

**Step 4 (recruit a new place cell).**

First, VPCs' firing rates are compared with each other. Then, the winner is compared with the given FRT. If the winner is below FRT, current place code is memorized, and a new VPC is recruited to be associated with this place code.

Through the above four steps, the vehicle can generate VPCs during its spatial exploration. Next, the proposed method will be validated and analyzed by simulation.

## 3. Results and Analysis

### 3.1. Realization of the Method

**Figure 4:**
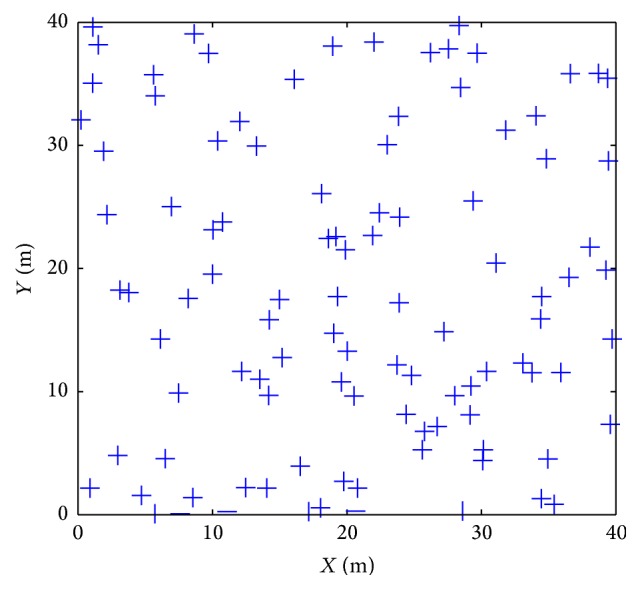
Landmarks in the given space.

The simulation conditions are set as follows:

(1) The vehicle's running environment is defined in a rectangular space, and its size is set to 40 m × 40 m. There are one hundred random distribution landmarks in the given space, as shown in Figure 4.
(2) A recognition distance is set to select the landmarks which are associated with current vehicle's location. Its role is similar to the method used to acquire landmarks. Suppose that the vehicle can recognize the landmarks whose distance is between 10 m and 15 m relative to vehicle (in actual environment, this condition can be removed, and the acquisition of landmarks is implemented by specific algorithm). Besides, the saliency values of selected landmarks are equal.
(3) The vehicle runs randomly in the defined space. Its maximal running speed is set to 5 m/s, and the location updating period is set to 1 s. Vehicle' speed remains unchanged in each period, but it changes randomly in different periods. Besides, the running direction is changed when the vehicle arrives at the boundary, and the relation between the new direction and the original direction obeys the reflection theorem. For the single spatial exploration, the total location updating step is set to 4000.
(4) *w*_*d*_ and *w*_*θ*_ are set to 1. *σ*_*d*_^2^ is set to 25. *σ*_*θ*_^2^ is set to 100. FRT is set to 0.2.

**Figure 5:**
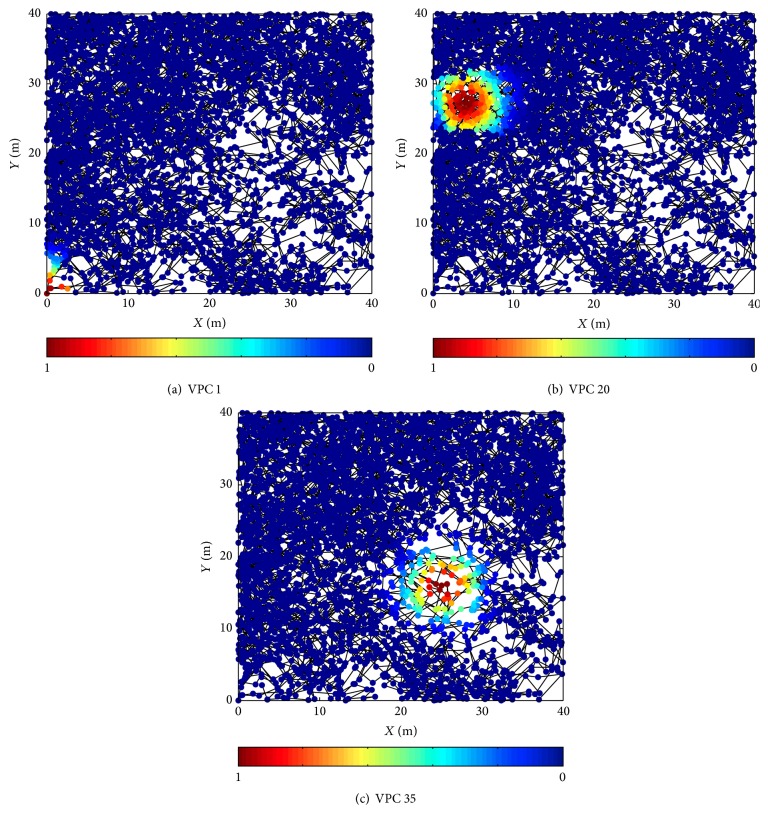
Firing status of partial VPCs.

**Figure 6:**
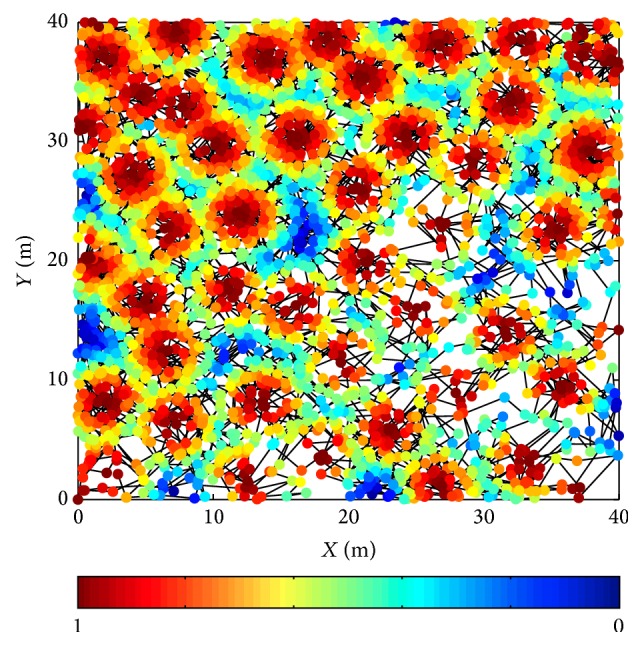
Firing status after the overlapping of the whole firing fields (generated VPCs' number is 50).

Figure 5 shows the firing status of partial VPCs after a single spatial exploration.  Figure 6 shows the firing status after the overlapping of the whole generated firing fields. For the simulation results, vehicle's trajectory is indicated by black line. The firing rate indicated by dark red and dark blue is corresponding to the highest and lowest firing rate, respectively. Simulation results show that the behavior of the generated VPCs is similar to that of biological place cells. A single VPC's firing field is corresponding to a restricted region, and the overlapping of the firing fields of the whole VPCs can cover the entire space. Therefore, the proposed method is available. The generated VPCs can well simulate the firing activity of biological place cells.

### 3.2. Influence on VPCs from the Parameters

Next, the influence on VPCs from AFFF, FRT, recognition distance, and location updating step will be discussed.

**Figure 7:**
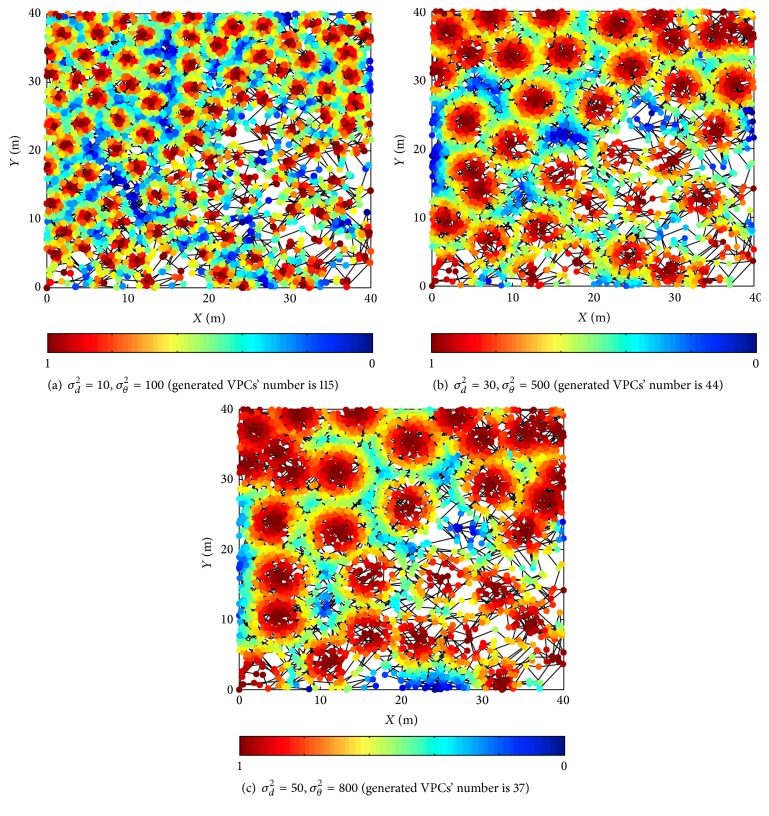
Firing status of VPCs in different AFFFs.

Figure 7 shows the firing status of VPCs in different AFFFs. Each simulation is carried out at the same trajectory. The other parameters are set according to the simulation conditions of Section 3.1. Simulation results show that AFFF affects the firing status of generated VPCs. When the AFFF increases, the single VPC's firing field extends and simultaneously VPCs' number used to represent the spatial environment decreases. That is to say, the setting of AFFF can affect the spatial representation precision. The smaller the AFFF, the higher the representation precision, but it also increases the memory cost. Therefore, the AFFF can be set flexibly according to the demand of representation precision and memory cost.

**Figure 8:**
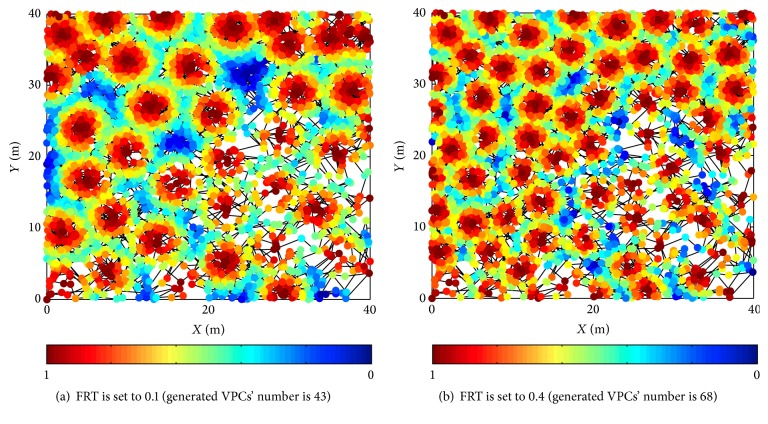
Firing status of VPCs in different FRTs.

**Figure 9:**
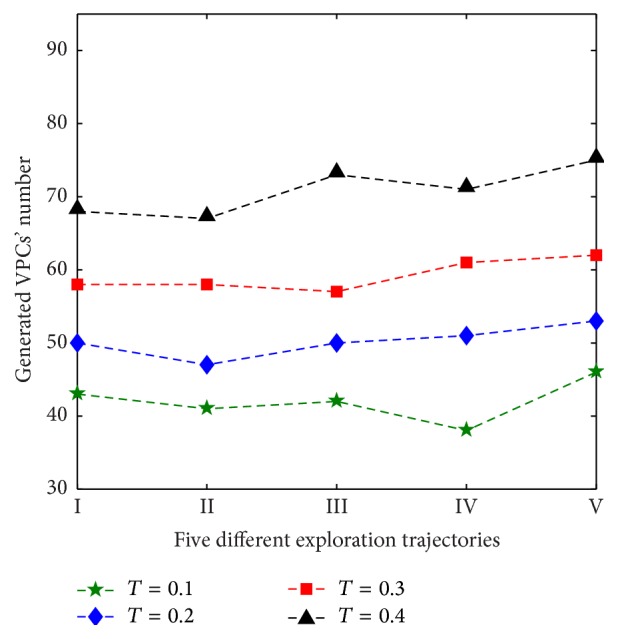
Generated VPCs' number in different FRTs (FRT is denoted by *T*).

Figures 8 and 9 show the firing status of VPCs and the generated VPCs' number in different given FRTs, respectively. FRT is divided into four different values, including 0.1, 0.2, 0.3, and 0.4. Five different exploration trajectories are implemented in the same FRT. The other parameters are set according to the simulation conditions of Section 3.1. Simulation results show that FRT affects VPCs' firing field and the generated VPCs' number. The bigger the FRT, the smaller the single firing field, and simultaneously the generated VPCs' number increases. Thus, the spatial representation precision and the memory cost can also be adjusted by setting different FRTs.

**Figure 10:**
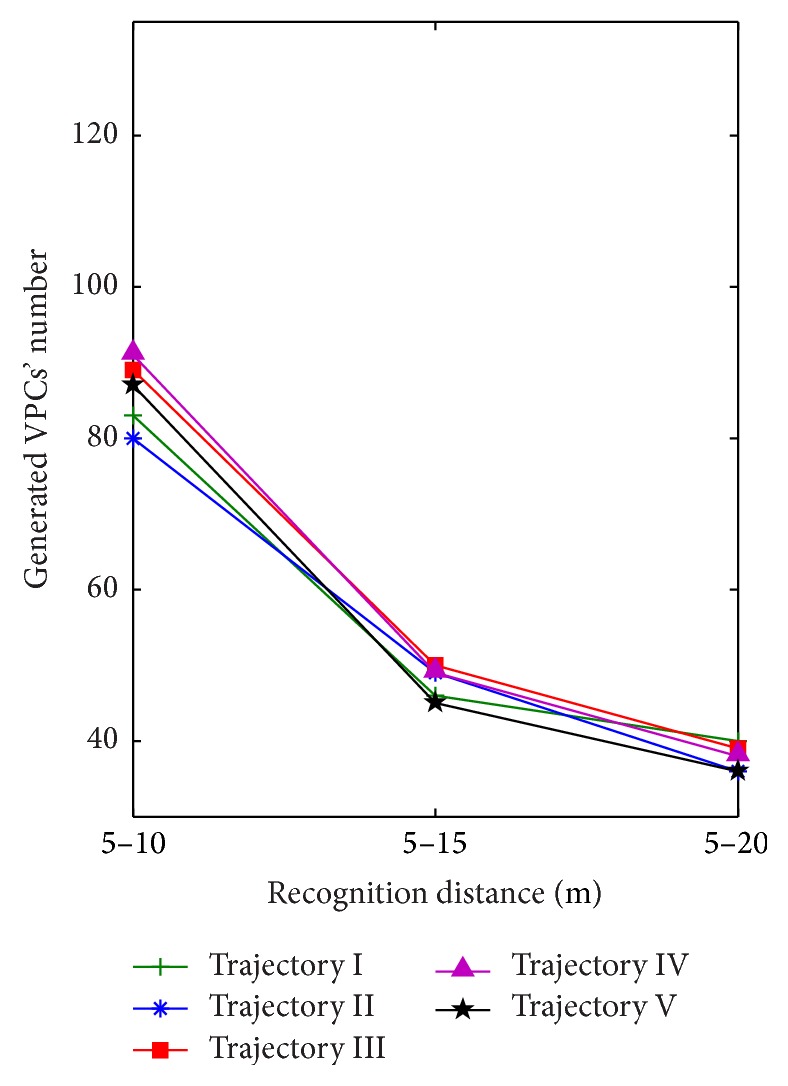
Generated VPCs' number in different recognition distances.

Figure 10 shows the generated VPCs' number in different recognition distances. The recognition distance is divided into three different intervals, including 5 m–10 m, 5 m–15 m, and 5 m–20 m. Five different exploration trajectories are implemented in the same recognition distance. The other parameters are set according to the simulation conditions of Section 3.1. Simulation results show that the recognition distance affects the generated VPCs' number. The longer the recognition distance, the less the generated VPCs' number. What is more, the number of obtained landmarks increases with the increasing of the recognition distance, so we can also get that the generated VPCs' number will decrease when more landmarks are used to calculate VPCs' firing rates.

**Figure 11:**
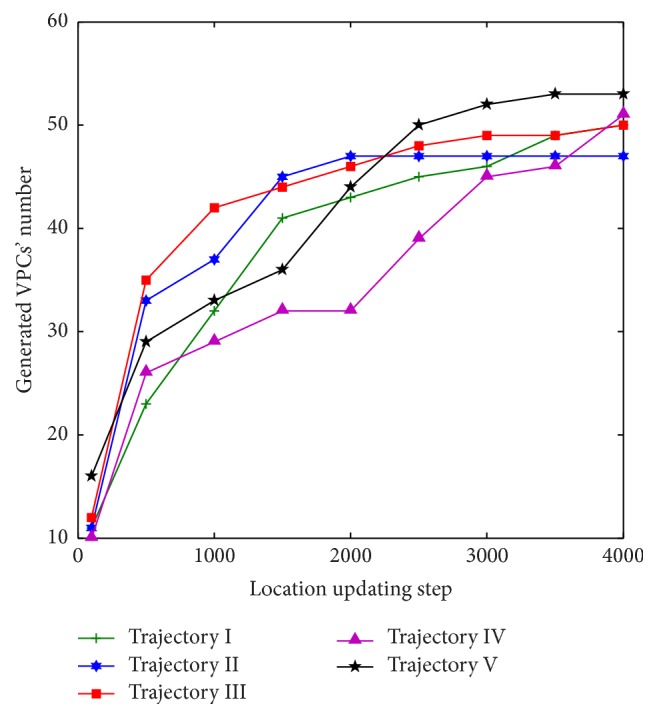
Generated VPCs' number in different location updating steps.

Figure 11 shows the generated VPCs' number in different location updating steps. Five different exploration trajectories are implemented in the same condition. The other parameters are set according to the simulation conditions of Section 3.1. Simulation results show that the more the location updating step, that is to say, the more the complete exploration of the environment, the more the generated VPCs' number, but the differences of generated VPCs' number will decrease or disappear when the higher number of location updating steps is implemented.

## 4. Conclusions

Combining the existing method to generate VPCs, this paper abstracts a model of generating VPCs based on environment perception and similar measure. In the model, the acquisition and representation of landmarks are included in the environment perception, and the calculation of VPCs' firing rates is included in the similarity measure, which can provide clear and complete process to generate VPCs. Simulation results show that the firing characteristic of generated VPCs is similar to that of biological place cells. VPCs' firing fields are corresponding to local regions, and the overlapping of the whole firing fields can cover the explored space. Simultaneously, the firing fields and the generated VPCs' number can be adjusted by setting different AFFFs and FRTs. In the next researches, we will discuss the proposed model in the actual environment and especially analyze the influence on the generation results of VPCs from landmarks' distribution and saliency value. In the actual environment, the distribution of landmarks is usually complex. For some locations, a lot of landmarks may be perceived by the vehicle, but, for other locations, the perceived landmarks may be very few. Besides, the landmarks' saliency values should be calculated by specific algorithm.
